# Coinfection with SARS-CoV-2 and Cytomegalovirus in a Patient with Mild COVID-19

**DOI:** 10.1155/2023/6684783

**Published:** 2023-05-30

**Authors:** Kazuya Ura, Yumi Goubaru, Misato Motoya, Hidehiro Ishii

**Affiliations:** ^1^Department of General Internal Medicine, Saiseikai Futsukaichi Hospital, Fukuoka, Japan; ^2^Department of Diabetology, Saiseikai Futsukaichi Hospital, Fukuoka, Japan

## Abstract

Persistent fever due to coronavirus disease 2019 (COVID-19) is a considerable issue for patients and physicians that requires a broad differential diagnosis and evaluation of complications. Coinfections with severe acute respiratory syndrome coronavirus 2 (SARS-CoV-2) and various respiratory viruses have also been reported. In severe cases of COVID-19, cytomegalovirus (CMV) reactivation or CMV coinfection with SARS-CoV-2 has been reported in association with critical illnesses and immunosuppressive therapy; however, in mild COVID-19 cases, CMV coinfection with SARS-CoV-2 has been reported only in severely immunocompromised patients, and its incidence and clinical importance remain unclear. Herein, we report a rare case of coinfection with SARS-CoV-2 and CMV in a patient with mild COVID-19 and untreated diabetes mellitus, which led to persistent fever for approximately 4 weeks. CMV coinfection should be considered in patients with COVID-19 who exhibit persistent fever.

## 1. Introduction

Severe acute respiratory syndrome coronavirus 2 (SARS-CoV-2) causes coronavirus disease 2019 (COVID-19), which continues to threaten global health [1]. Safe and effective vaccines and treatment options are now available [2]; however, COVID-19 still causes hospitalization or death, especially in the elderly and in people with pre-existing conditions. Persistent fever in patients with COVID-19 is of great concern to physicians and patients and requires a thorough evaluation for complications, such as pneumonia, thrombosis, or myocardial injury [3]. This may result in unnecessary antibiotic prescriptions.

Cytomegalovirus (CMV), a member of the *Herpesviridae* family, is ubiquitous in humans. CMV infection causes severe disease in immunocompromised patients; however, it can cause long-term fever in healthy adults due to infectious mononucleosis (IM) syndrome [4]. In many countries, the CMV seroprevalence rates are decreasing among young adults and children, particularly in developed countries, and these populations are at a higher risk of primary CMV infection [5]. Recently, CMV infection has been associated with severity or mortality of COVID-19 [6, 7]. In severe COVID-19 cases, CMV reactivation or CMV and SARS-CoV-2 coinfection is a major complication associated with immunosuppressive therapy [8–10]. However, in mild COVID-19 cases, such as those without pneumonia or respiratory failure, there have been only a few reports of coinfection with SARS-CoV-2 and CMV in immunocompromised patients.

Herein, we describe a rare case of coinfection with SARS-CoV-2 and CMV that caused persistent fever in a patient diagnosed with mild COVID-19. Accurate diagnosis of CMV and SARS-CoV-2 coinfection as the cause of persistent fever in COVID-19 would benefit patients and clinicians by preventing unnecessary examinations and antimicrobial prescriptions.

## 2. Case Report

A 36-year-old man presented at our hospital complaining of a fever persisting for approximately 2 weeks. Three weeks before admission, he became febrile and was diagnosed with COVID-19 by polymerase chain reaction (PCR) testing of a nasopharyngeal swab. The following week, he developed a persistent low-grade fever while reposing at home, and then he developed a high-grade fever of higher than 39.0°C, along with mild headache, nausea, and anorexia. Two days prior to admission, he visited the emergency department of our hospital; however, chest computed tomography (CT) scans were negative for pneumonia, and the patient was prescribed acetaminophen. On the day of admission, he presented to our department for further evaluation.

Approximately 4-5 years earlier, he was diagnosed with diabetes mellitus (DM), for which he had not sought any medical attention, and he occasionally consumed alcohol but did not smoke. Prior to the presentation, his wife and daughter were diagnosed with COVID-19. He had not traveled nor been exposed to any animals recently. He was not prescribed any medication and had no known allergies. He had received two doses of a messenger RNA SARS-CoV-2 vaccine.

Upon examination, the patient was alert and did not experience acute distress. The patient's blood pressure was 118/83 mmHg, heart rate was 84 beats per minute, axillary temperature was 36.7°C, and SpO_2_ reading was 97% while breathing ambient air. No jolt accentuation or cervical lymphadenopathy was observed. Chest auscultation revealed no murmurs or rales. There was mild epigastric tenderness in the left upper quadrant, but no hepatosplenomegaly and no eruptions are observed.

**Figure 1:**
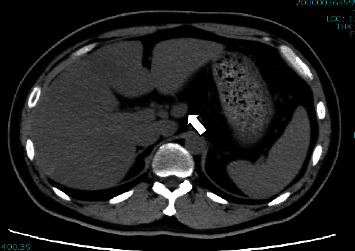
Plain abdominal CT on day 2 of hospitalization showed mild splenomegaly (arrowhead) and intra-abdominal lymphadenopathies (arrow).

Upon admission, a SARS-CoV-2 antigen test of the nasopharyngeal swab was negative at 0.83 pg/mL. The patient's white blood cell (WBC) count was 8000/*μ*L, with 29% neutrophils and 57% lymphocytes; however, no atypical lymphocytes were observed. Liver function test results revealed aspartate aminotransferase (AST), alanine aminotransferase (ALT), and total bilirubin (T-Bil) levels of 40 U/L, 68 U/L, and 0.9 mg/dL, respectively. The patient's blood urea nitrogen (BUN) and creatinine levels were 10.9 mg/dL and 0.59 mg/dL, respectively. Serum C-reactive protein (CRP) and procalcitonin levels were 7.81 mg/dL and 0.19 ng/mL, respectively. Hemoglobin A1c and blood glucose levels were 10.9% and 322 mg/dL, respectively. The other laboratory test results are presented in Table 1. Abdominal CT without contrast revealed mild splenomegaly, mild fatty liver, and intra-abdominal lymphadenopathy suggestive of inflammatory swelling (Figure 1).

The patient was admitted, and acetaminophen was administered orally and intravenously, which partially relieved his fever, fatigue, and headache. Blood and urine cultures were negative and no antimicrobials were prescribed; thus, we suspected a viral infection and performed serological screening for CMV, Epstein–Barr virus (EBV), herpes simplex virus, and human immunodeficiency virus (HIV). The patient's CMV immunoglobulin M (IgM) level was elevated, and other tests showed negative results or signs of a past infection. Given the possibility of primary CMV infection, we proceeded with further evaluations, which revealed a positive status for CMV antigenemia (C7-HRP, 80/50000 WBCs) and a CMV DNA PCR level of 7800 IU/mL (Table 2). Visual inspection of the peripheral blood on day 4 revealed an atypical lymphocyte percentage of 21.0% (WBC 7800/*μ*L). The patient was diagnosed with IM due to primary CMV infection. The patient's HIV antibody and p24 antigen test results were negative, and the CD4 count was 764/*μ*L. The fever disappeared within 10 days, with remission of all symptoms, without any specific antiviral treatment. During admission, his DM was well controlled by treatment, initially with insulin and then with an oral hypoglycemic agent. The patient was discharged 15 days after admission.

## 3. Discussion

Here, we present a rare case of persistent fever due to CMV and SARS-CoV-2 coinfection in a patient with mild COVID-19. The incubation period for a CMV infection is about 4 to 6 weeks; this indicates that the patient was infected with CMV when he was diagnosed with COVID-19. CMV and SARS-CoV-2 coinfection should be considered as a differential diagnosis for patients with COVID-19 who present unexplained persistent fever.

Persistent fever in COVID-19 has led to the identification of complications such as pneumonia; however, after the Omicron surge, when the incidence of pneumonia and respiratory failure were low, it became important to evaluate the cause of fevers associated with COVID-19 and those unrelated to COVID-19. The Omicron variant was the dominant SARS-CoV-2 strain in Japan when the present patient was infected with SARS-CoV-2 in August 2022. Thus, we presumed this patient was infected with the Omicron variant, although the genotype of the virus was not assessed.

CMV infection has been associated with severity or mortality of COVID-19 [6, 7]. Most reports on CMV coinfection with SARS-CoV-2 are limited to severe cases, such as patients treated with systemic glucocorticoids and invasive mechanical ventilation in the intensive care unit setting [8–10]. These reports were obtained during the early phase of the SARS-CoV-2 pandemic (to mid-2022) when the mortality rate was higher than that of the current pandemic (after late 2022, the Omicron phase). Although these reports [8–10] did not include SARS-CoV-2 patient vaccination data, a rare case of SARS-CoV-2 coinfection with CMV reactivation in an immunocompromised patient who received the SARS-CoV-2 vaccination was also reported [11].

Coinfections with SARS-CoV-2 and other pathogens, such as viruses, bacteria, and fungi, have been reported; however, most of these viruses are respiratory [12–16]. Few cases of CMV coinfection with SARS-CoV-2 in mild COVID-19 (i.e., without pneumonia or respiratory failure) have been reported in immunocompromised patients, such as those with acquired immunodeficiency syndrome, hematologic malignancy, kidney transplant recipient, or a pregnant woman [17–20]. CMV coinfection with SARS-CoV-2 in mild COVID-19 cases in patients without serious medical conditions is very rare, and its incidence and clinical significance remain unclear.

CMV infection is usually asymptomatic, but it may cause IM syndrome in healthy adults and severe disease in neonates and immunocompromised patients. Here, the patient had only nonspecific symptoms, such as abdominal discomfort, nausea, and a slight headache, which are mainly associated with fever. Compared with IM due to EBV, CMV-IM is reportedly less likely to induce typical symptoms, such as pharyngitis with exudates, cervical lymphadenopathy, or hepatosplenomegaly [21]. Therefore, it may be difficult to suspect a CMV infection based on clinical symptoms, and this infection cannot be diagnosed without performing specific testing for viral markers.

Furthermore, CMV antigenemia, an assay for detecting the PP65 antigen (C7-HRP), is not routinely used to evaluate for CMV infections in immunocompetent patients. However, CMV-IgM reportedly exhibits cross-reactivity with other viral pathogens [22], a prolonged elevation 1 year after the onset of infection, and an elevation in reactivated CMV infection; thus, we performed an antigenemia and PCR to confirm CMV infection in this case.

Recently, the seroprevalence rates of CMV have decreased in young adults and children, particularly in the developed countries [5]. Thus, the range of populations susceptible to CMV is increasing worldwide. As long as the COVID-19 pandemic continues, similar cases of COVID-19 presenting persistent fever without COVID-19-associated sequelae, such as pneumonia, may increase. The accurate diagnosis of CMV infection in these cases would be beneficial for patients and clinicians.

Several reports have described an association between CMV and SARS-CoV-2 infection. Both viruses influence the immune system [6] and may have a detrimental effect on the course of these infections. High CMV-seropositivity is associated with severe COVID-19 [7], possibly due to CMV association with the acceleration of immune senescence and cardiovascular and metabolic diseases [9]. Furthermore, in patients with severe COVID-19, the reactivation of CMV has been associated with high mortality rate; however, little is known about the interactions between primary CMV and SARS-CoV-2 infections, as in the present case. It is unknown whether simultaneous primary CMV and SARS-CoV-2 infections are risk factors for severe COVID-19, and vice versa. In addition, there is a reported case of CMV reactivation after COVID-19 vaccination [23]; however, further reports on these cases and accumulation of knowledge are needed.

Here, the patient had DM, which causes immune dysfunction and is a recognized risk factor for severe COVID-19; however, DM has not been reported as a risk factor for CMV infection. The patient in our case had a persistent fever for approximately 4 weeks, which was longer than that previously reported by a mean of 18 days [24], and a markedly high level of antigenemia. It is possible that the untreated DM influenced the patient's clinical course.

In conclusion, we present a case of CMV coinfection in a patient with mild COVID-19. CMV infection screening in patients with COVID-19 who have unexplained persistent fever is important as it prevents unnecessary evaluations or antibiotic prescription and is beneficial to patients and physicians.

## Figures and Tables

**Table 1:** Laboratory examinations on admission.

*Complete blood count*		
WBC	8000	*μ*L
Neutrophils	29.0	%
Lymphocytes	57.0	%
Monocytes	11.0	%
Eosinophils	3.0	%
Basophils	—	%
Atypical lymphocytes	—	%
Ht	44.5	%
RBC	548	×10^4^/*μ*l
Hb	15.9	g/dL
Plt	19.8	×10^4^/*μ*l

*Biochemical examinations*		
TP	7.1	g/dL
Alb	3.6	g/dL
BUN	10.1	mg/dL
Crea	0.59	mg/dL
T-Bil	0.9	mg/dL
AST	40	U/L
ALT	68	U/L
*γ*GTP	40	U/L
ALP	108	U/L
LDH	281	U/L
CK	26	U/L
CRP	7.81	mg/dL
PCT	0.19	ng/mL
Glu	322	mg/dL
HbA1c	10.9	%

*Immunology and infection*		
IgM	95	mg/dL
IgG	1329	mg/dL
IgA	429	mg/dL
C3	178	mg/dL
C4	46	mg/dL
CH50	61.2	mL
HBs-Ag	0.01	IU/mL
HCV-Ab	0.02	C.O.I
EBV-VCA IgM (FAT)	<10.0	
EBV-EBNA (FAT)	40	
HSV IgM (EIA)	0.25	
HIV Ag/Ab (CLEIA)	(−)	
CD4	764	/*μ*L
CD4/CD8	0.35	

WBC, white cell count; Ht, hematocrit; RBC, red cell count; Hb, hemoglobin; Plt, platelet count; PT, prothrombin time; PT-INR, prothrombin time-international normalized ratio; APTT, activated partial thromboplastin time; FDP, fibrin/fibrinogen degradation products; TP, total protein; Alb, albumin; BUN, blood urea nitrogen; Cre, creatinine; T-Bil, total bilirubin; AST, aspartate aminotransferase; ALT, alanine aminotransferase; *γ*GTP, gamma-glutamyl transpeptidase: ALP, alkaline phosphatase; LDH, lactate dehydrogenase; CK, creatine kinase; CRP, C-reactive protein; PCT, procalcitonin; Glu, glucose; HbA1c, hemoglobin A1c; IgM, immunoglobulin M; IgG, immunoglobulin G; IgA, immunoglobulin A; HBs-Ag, hepatitis B surface antigens; HCV-Ab, hepatitis C antibody; EBV, Epstein–Barr virus; VCA, viral capsid antigen; FAT, fluorescent antibody technique; EBNA, Epstein–Barr virus nuclear antigen; HSV, herpes simplex virus; EIA, enzyme immunoassay; HIV, human immunodeficiency virus; Ag/Ab, antigen/antibody; and CLEIA, chemiluminescent enzyme immunoassay.

**Table 2:** Time course of CMV marker in the present case.

CMV marker	Day 3	Day 1 (admission)	Day 4	Day 8	Day 14
CMV-IgM (CLIA)	2.82	—	—	4.67	4.45
CMV IgG (CLIA)	8.5	—	—	75.4	83.3
CMV antigenemia	—	80/50000	—	4/50000	4/50000
CMV DNA (PCR)	—	—	7800 IU/mL	—	—

CMV, cytomegalovirus; IgM, immunoglobulin M; CLIA, chemiluminescent immunoassay; IgG, immunoglobulin G; IgA, immunoglobulin A; DNA, deoxyribonucleic acid; PCR, polymerase chain reaction.

## Data Availability

The data supporting the findings of this study are available within the article.

## Abbreviations

SARS-CoV-2: Severe acute respiratory syndrome coronavirus 2
COVID-19: Coronavirus disease 2019
CMV: Cytomegalovirus
IM: Infectious mononucleosis
PCR: Polymerase chain reaction
CT: Computed tomography
DM: Diabetes mellitus
WBC: White blood cell
AST: Aspartate aminotransferase
ALT: Alanine aminotransferase
T-Bil: Total bilirubin
BUN: Blood urea nitrogen
CRP: C-reactive protein
EBV: Epstein–Barr virus
HIV: Human immunodeficiency virus
IgM: Immunoglobulin M.
